# Structural bases for blockade and activation of BK channels by Ba^2+^ ions

**DOI:** 10.3389/fmolb.2024.1454273

**Published:** 2024-09-17

**Authors:** Shubhra Srivastava, Pablo Miranda, Teresa Giraldez, Jianghai Zhu, Raul E. Cachau, Miguel Holmgren

**Affiliations:** ^1^ Molecular Neurophysiology Section, National Institute of Neurological Disorders and Stroke, National Institutes of Health, Bethesda, MD, United States; ^2^ Institute of Biomedical Technologies & Department Basic Medical Sciences, School of Medicine, University of La Laguna, Tenerife, Spain; ^3^ Integrative Data Science Section, Research Technologies Branch, National Institute of Allergy and Infectious Diseases, Bethesda, MD, United States

**Keywords:** membrane, voltage, divalent, binding site, RCK domain

## Abstract

We studied the impact of Ba^2+^ ions on the function and structure of large conductance potassium (BK) channels. Ion composition has played a crucial role in the physiological studies of BK channels due to their ability to couple ion composition and membrane voltage signaling. Unlike Ca^2+^, which activates BK channels through all *Regulator of K* ^*+*^ *Conductance* (RCK) domains, Ba^2+^ has been described as specifically interacting with the RCK2 domain. It has been shown that Ba^2+^ also blocks potassium permeation by binding to the channel’s selectivity filter. The Cryo-EM structure of the *Aplysia* BK channel in the presence of high concentration Ba^2+^ here presented (PDBID: 7RJT) revealed that Ba^2+^ occupies the K^+^ S3 site in the selectivity filter. Densities attributed to K^+^ ions were observed at sites S2 and S4. Ba^2+^ ions were also found bound to the high-affinity Ca^2+^ binding sites RCK1 and RCK2, which agrees with functional work suggesting that the Ba^2+^ increases open probability through the Ca^2+^ bowl site (RCK2). A comparative analysis with a second structure here presented (PDBID: 7RK6), obtained without additional Ba^2+^, shows localized changes between the RCK1 and RCK2 domains, suggestive of coordinated dynamics between the RCK ion binding sites with possible relevance for the activation/blockade of the channel. The observed densities attributed to Ba^2+^ at RCK1 and RCK2 sites and the selectivity filter contribute to a deeper understanding of the structural basis for Ba^2+^'s dual role in BK channel modulation, adding to the existing knowledge in this field.

## Introduction

The voltage-dependent and Ca^2+^-activated potassium (BK) channels are unique, given their large single-channel conductance, allowing a detailed characterization of their properties. BK channels couple Ca^2+^ with membrane voltage signaling, playing essential roles in various physiological functions (Latorre et al., 2017). BK channels are tetramers forming a large intracellular structure with eight high-affinity Ca^2+^ sites (Hite et al., 2017; Tao et al., 2017) called the gating ring (Jiang et al., 2002). Each Ca^2+^ site is embedded in a regulator of potassium conductance (RCK) domain (Jiang et al., 2001). Ca^2+^ ions interact with BK channels’ RCK1 and RCK2 domains. *Aplysia* BK channels Ca^2+^ bowl site uses two side-chain carboxylates (Asp905 and Asp907) (Hite et al., 2017; Tao et al., 2017) and two main-chain carboxyl oxygens (Gln899 and Asp902) of the RCK2 domain, and the side-chain of Asn438 from the RCK1 domain of the neighboring subunit to coordinate a Ca^2+^ ion. Ca^2+^ at the high-affinity site of the RCK1 domain is interacting with two side-chain carboxylates (Asp356 and Glu525) and three main-chain carboxylates (Glu591, Arg503, Gly523). Many divalent ion species (Mn^2+^, Ni^2+^, Mg^2+^, and Co^2+^) do not activate BK channels through the high-affinity binding sites (Zeng et al., 2005), while Sr^2+^, as Ca^2+^, can activate BK channels through the RCK1 and the Ca^2+^ bowl sites (Zeng et al., 2005). Cd^2+^ activates through the RCK1 domain (Zeng et al., 2005; Zhang et al., 2010). Ba^2+^ properties are more complex. Ba^2+^ has been described as a potent blocker of BK channels (Vergara and Latorre, 1983), and detailed kinetic studies at the single-channel level provided consistent evidence that blockade likely occurred within the narrowest region of the permeation pathway. Access of Ba^2+^ from the inside and the outside to the blocking site is voltage-dependent (Vergara and Latorre, 1983). Increasing K^+^ concentration diminishes Ba^2+^ on-rate to its site (Vergara and Latorre, 1983). Access of Ba^2+^ to its location is through an open channel (Miller et al., 1987), and closing the channel with a Ba^2+^ bound can trap the blocker for minutes (Miller, 1987). By a thorough study of the influences of intracellular and extracellular K^+^ on the kinetics of Ba^2+^ blockade (Neyton and Miller, 1988a; b), it was proposed that the Ba^2+^ blocking site is wrapped between two K^+^ ions lock-in sites in the narrow region of the permeation pathway. More recently, Ba^2+^ was also found to activate BK channels through the Ca^2+^ bowl site (Zhou et al., 2012; Miranda et al., 2016), yet a unified model integrating the functional properties of Ba^2+^ has yet to be obtained. Here, we present two cryoEM structures of *Aplysia* Slo1 BK channels with Ba^2+^ incorporated into nanodiscs. These structures reveal that Ba^2+^ can occupy nine sites within the BK channel tetrameric complex, eight in the gating ring, and one at site S3 of the selectivity filter. Because these structures were obtained with BK channels exposed to different concentrations of Ba^2+^, comparative analysis revealed differences at the gating ring and the selectivity filter, providing details of the mechanisms involved in blockade and activation.

## Methods

### Expression, purification, and reconstitution of *Aplysia* Slo1

The full-length Slo1 gene from *Aplysia californica* was expressed in Sf9 cells with a C-terminal GFP/rho-1D4 tag cleavable by precision protease. Baculovirus was generated in Sf9 cells using the Bac-to-Bac system. Cells were harvested and lysed, and membranes were collected by centrifugation. The membrane pellet was homogenized and treated with DDM and CHS for protein extraction. After ultracentrifugation, the supernatant was affinity purified using a GFP nanobody resin. The protein was cleaved and further purified by size-exclusion chromatography. Slo1 tetramers were incorporated into lipid nanodiscs with MSP1E3D1 (see Supplementary Material and method section).

### CryoEM

Samples were prepared following the standard workflow for cryoEM SPA. Grids were vitrified using a Vitrobot MK IV (Thermo Fisher Scientific). CryoEM data were collected on a Titan Krios electron microscope (Thermo Fisher Scientific) with a K2 detector (Gatan) and processed using cryoSPARC (Punjani et al., 2020) and RELION (Scheres, 2012). *Aplysia* Slo1 structure (PDBID:5tj6) was docked into maps using ChimeraX (Pettersen et al., 2021). Models were built in COOT (Emsley and Cowtan, 2004) and refined in PHENIX.real_space_refine (Adams et al., 2010). See Supplementary Material for a more details.

### Electrophysiological characterization of Ba^2+^ dual-functional effects on BK channels

Full-length Slo1 from *Aplysia* was subcloned into the pGemHE vector (Liman et al., 1992). Next, the plasmid was linearized with NheI before performing RNA *in vitro* T7 polymerase transcription with Ambion, Thermo Fisher Scientific kit. Finally, 50 ng of synthesized RNA was injected into each *Xenopus laevis* oocyte. Oocytes were purchased from Ecocyte Bioscience. Excised inside-out patches were obtained using borosilicate pipettes (VWR 53432–921) with tips ranging between 0.7–1 MΩ, using recording solutions containing (in mM): pipette, 40 KMeSO_3_, 100 N-methylglucamine–MeSO_3_, 20 HEPES, 2 KCl, 2 MgCl_2_, 100 μM CaCl_2_ (pH = 7.4); bath solution, 40 KMeSO_3_, 100 N-methylglucamine–MeSO_3_, 20 HEPES, 2 KCl, 1 EGTA, and free BaCl_2_ concentrations varying between 1–100 µM, previously estimated using Maxchelator (Bers et al., 1994). Currents were recorded with an Axopatch 200B amplifier using Clampex software (Axon Instruments, Molecular Devices). Solutions containing different ion concentrations were exchanged using a fast solution exchange (BioLogic RSC-200).

## Results

### Activation and blockade of *Aplysia* Slo1 by Ba^2+^

**FIGURE 1:**
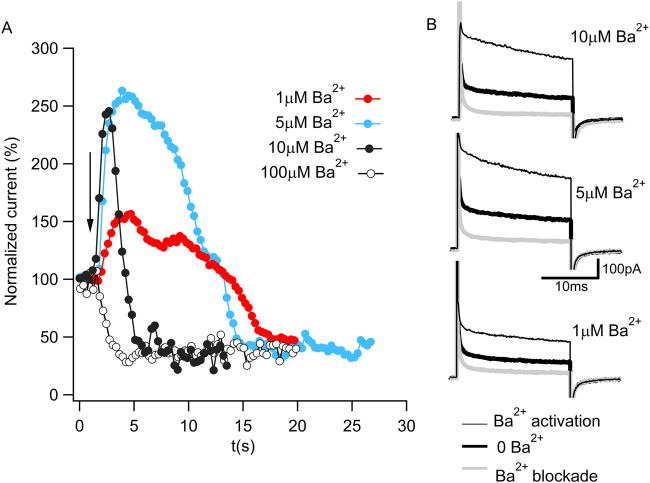
Ba^2+^ activation and blockade of BK current. **(A)** Time course of normalized K^+^ currents in response to 1 μM (red), 5 μM (cyan), 10 μM (full black circle) or 100 μM (open circle) Ba^2+^ applied to an inside-out patch containing *Aplysia* BK channels. Each dot corresponds to the average current of the last 10 ms of a 20 ms pulse to 120 mV from a holding potential of −70 mV in 300 ms intervals. The arrow represents the moment of the Ba^2+^ addition. **(B)** Representative current traces in 10 μM Ba^2+^ (top panel), 5 μM Ba^2+^ (middle panel) and 1 μM Ba^2+^ (bottom panel) from Figure 1A. The current in the absence of Ba^2+^ (bold black trace) increases during Ba^2+^ activation (thin black trace) and reduces after Ba^2+^ blockade (gray trace).

Ba^2+^ was initially reported to be a potent BK channel blocker (Vergara and Latorre, 1983). However, within the last decade, Ba^2+^ has also been shown to activate BK channels (Zhou et al., 2012; Miranda et al., 2016). To monitor these two functional properties of Ba^2+^ in *Aplysia* Slo1 channels, we recorded macroscopic currents using excised inside-out patches and repeatedly applied 20-ms voltage steps to +120 mV from a holding potential of −70 mV at a duty cycle of 1 pulse per 300 ms. Figure 1A shows the steady-state normalized current before and after (arrow) four different Ba^2+^ concentration exposures to the same patch. In the presence of 100 µM Ba^2+^, a fast blockade of the permeation pathway is observed (open circles). However, exposing the channels to lower Ba^2+^ concentrations, the blockade is slower, unveiling activation by Ba^2+^ that preceded the block (black, cyan, and red circles for 10, 5, and 1 µM Ba^2+^, respectively). Figure 1B shows representative current traces before Ba^2+^ activation (thick black traces), during activation (thin black traces), and after blockade (thick gray traces) for exposures to 10, 5, and 1 µM Ba^2+^. These results confirm that Ba^2+^ can block and activate *Aplysia* Slo1 with similar features as the human Slo1 (Zhou et al., 2012; Miranda et al., 2016).

### Structures of *Aplysia* Slo1 in the presence of Ba^2+^

**FIGURE 2:**
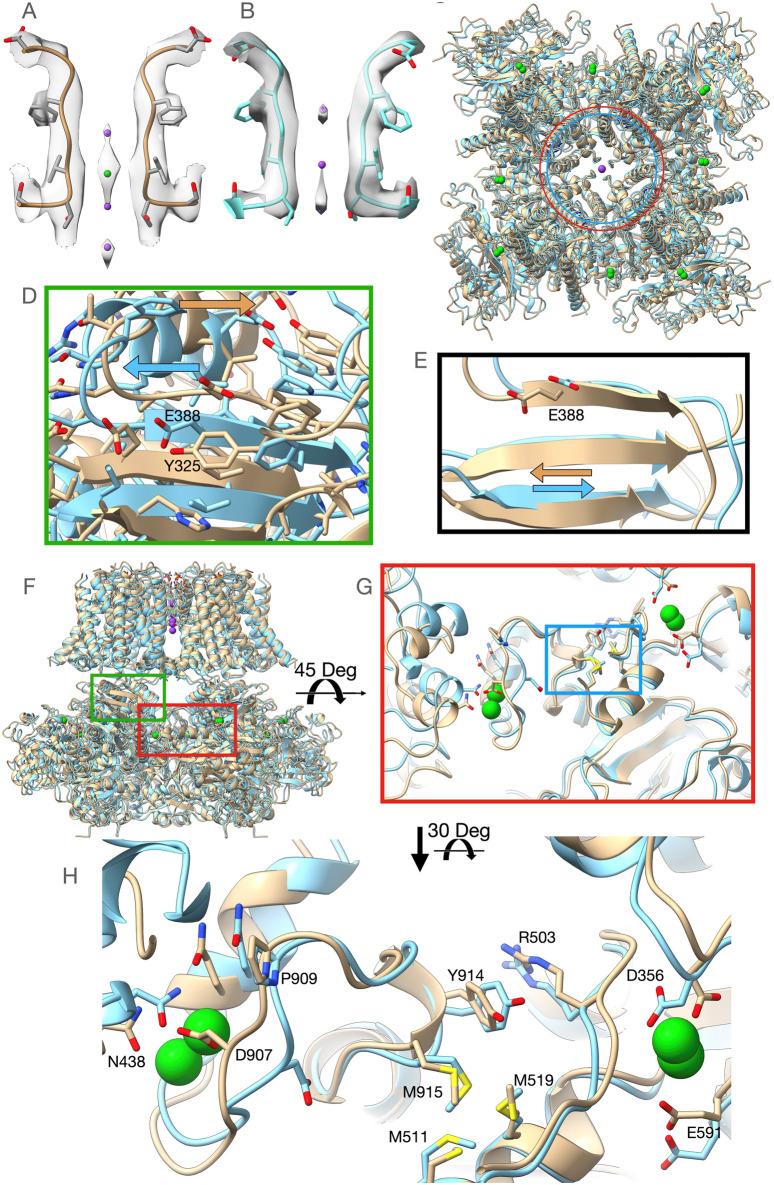
Overview of the High-Barium (light brown) and Low-Barium (light blue) structures. **(A)** The High-Barium structure has a density attributed to Ba^2+^ at site S3. K^+^ ions, in purple, at sites S2 and S4 and, a third one at the entrance of the selectivity filter from the intracellular side. **(B)** The selectivity filter of the Low-Barium structure shows densities attributed to K^+^ ions at sites S2, S3, and S4. **(C)** The distance between Lys320 Ca atoms across the pore axis (40.7 Å) is shown as a red circle for the Low-Barium structure and in blue (44.7 Å) for High-Barium structure. **(D)** Helix aB shifts towards its C-terminal end in the High-Barium structure (arrows). **(E)** Shift of the Glu388 within the BC strand (arrows). **(F)** General view of the BK channel structures, indicating regions of interest. Green box corresponding to Figure 2D. Red box area is presented in Figures 2G, H. **(G)** The methionine cluster is within the blue box. **(H)** RCK1 - RCK2 region in two views rotated 30 Deg to facilitate the visualization of contacts. See supplementary video for details.

Our first structure (PDBID: 7RK6) was obtained from *Aplysia* Slo1 channel protein purified in the presence of 40 mM Ba^2+^ to ensure the replacement of any remaining Ca^2+^ ions from the expression system, but after nanodisc incorporation, it was run through the size exclusion chromatography column preequilibrated with a buffer lacking any divalent ions. We will refer to this structure as the Low-Barium structure (∼1.4 mM Ba^2+^, see Supplementary Method Section). The Low-Barium structure helps us identify the initial Ba^2+^ binding geometry at low ion concentration, i.e., the number and locations of densities attributed to Ba^2+^ ions. A second structure was obtained by adding 10 mM Ba^2+^ throughout the purification process. We will refer to this structure as the High-Barium structure (PDBID: 7RJT). The overall arrangement of these new structures is similar to those previously published *Aplysia* BK channel structures (Hite et al., 2017; Tao et al., 2017). Eight sites with strong map densities within the gating rings, attributed to Ba^2+^ ions, can be seen in both structures. The High-Barium structure has an additional density attributed to Ba^2+^ within the selectivity filter at the traditional K^+^ site S3 (Figure 2A). In this structure, three densities can be attributed to K^+^ ions at sites S2 and S4 and, interestingly, a third one at the entrance of the selectivity filter from the intracellular side (Figure 2A). The selectivity filter of the Low-Barium structure only has densities attributed to K^+^ ions, which are observed at sites S2, S3, and S4 (Figure 2B).

### Influence of Ba^2+^ on *Aplysia* Slo1

In the Ca^2+^/Mg^2+^ structure, divalent ions introduce a new bend of the S6 helices at Gly302 (Hite et al., 2017; Tao et al., 2017). Since this residue is near the inner end of the selectivity filter, this conformational change is likely a consequential movement linked to the opening of the permeation gate in the *Aplysia* Slo1 channel. This bend at Gly302 induces an expansion of the RCK1 N-lobes of ∼14 Å (monitored at the Cα atom of Lys320) relative to the structural model in the presence of 1 mM EDTA (Hite et al., 2017; Tao et al., 2017). In the presence of Ba^2+^, our structural models do not show the bend at Gly302. Nonetheless, we do observe that the distance between Cα atoms of the Lys320 across the pore axis was 40.7 Å for the Low-Barium and 44.7 Å for High-Barium structures, respectively (Figure 2C). As previously (Hite et al., 2017; Tao et al., 2017), the RCK1 N-lobe showed much larger motions in our two structures than the RCK1 C-lobe. The helix αB appears to shift towards its C-terminal end in the High-Barium structure (Figure 2D). This movement is driven by the displacement of the intracellular domain relative to the trans-membrane domain with the helix αB in close proximity to the interphase between the two domains. As a result of this movement, one of the Mg^2+^ coordinating residues, Glu388 within the βC strand, moves towards the position found in the Ca^2+^/Mg^2+^ structure (Hite et al., 2017; Tao et al., 2017) (Figure 2E). These observations help explain previous functional data showing that the Mg^2+^ affinity is higher in open Slo1 channels than in closed ones (Shi and Cui, 2001) and that the ability of Mg^2+^ to enhance channel activation is by affecting the close→open transition (Zhang et al., 2001).

Contrary to the RCK1, comparative analysis of the RCK2 domain displayed little differences between our structural models.

### Ba^2+^ ions at the high-affinity divalent binding sites of the gating ring

Ba^2+^ activates BK channels by interacting with the Ca^2+^ bowl (Zhou et al., 2012; Miranda et al., 2016). Yet, the Low-Barium and High-Barium structures have eight densities within the gating ring. Most residues interacting with Ca^2+^ at the RCK1 and RCK2 sites in the Ca^2+^/Mg^2+^ structure (Tao et al., 2017) interact with Ba^2+^ (Figures 2F–H). In the Ca^2+^ bowl, the small linker between helices 3_10_ and αR, containing Asp907, is quite flexible. Consequently, the side chain of Asp907 does not face the Ca^2+^ bowl binding pocket in the Low-Barium structure while in the High-Barium structure, it does (Figures 2G, H). In the RCK1 binding site, all side chains coordinating Ba^2+^ remain in a similar orientation in both the Low-Barium and High-Barium structures. The movement of Asp907 in response to saturating Ba^2+^, triggers an intricate series of concerted changes suggesting coordinated dynamics of the two binding sites within the same subunit (Supplementary Video S1), as previously suggested (Hite et al., 2017; Tao et al., 2017). Key residues involved in this flexible corridor are Pro909, Tyr914, Arg503, the Methionine cluster involving Met511, Met 519, and Met915, and an Asn438 from a neighboring subunit (Figures 2G, H). Some of these residues have been previously identified in functional studies (Shi et al., 2002; Zhang et al., 2010; Bukiya et al., 2014; Li et al., 2018). This rearrangement is facilitated by the rotation of the methionine cluster between the two binding sites (Supplementary Video). Beneath the flexible corridor is a rigid beta-strand structure impeding changes in the direction perpendicular to the membrane (Figure 2G). The general architecture of this flexible corridor is maintained in the hBK channels (Tao and MacKinnon, 2019).

The two structures here presented suggest the selective nature of Ba^2+^ activation is not the result of the absence of Ba^2+^ at the RCK1 site, yet we may speculate that the contribution of Asp356 to the coordination of a divalent ion at the RCK1 site (Hite et al., 2017; Tao et al., 2017; Tao and MacKinnon, 2019) is crucial to trigger the conformational changes responsible for the activation of the gating ring through the RCK1 domain. This residue shows a rearrangement between our two structures, possibly compensatory of the Arg503 displacement and partial disengagement from the Ba^2+^ coordination sphere at low ion concentration, suggesting a complex molecular choreography connecting the two domains.

## Discussion

The interactions of Ba^2+^ ions with BK channels are unique among divalent ions because they activate the channel by increasing the open channel probability and block K^+^ permeation. Here we presented two cryoEM structures of *Aplysia* Slo1 BK channels with Ba^2+^ incorporated into nanodiscs, that integrate the dual functional properties of Ba^2+^ in BK channels. On the one hand, in the High-Barium structure, a density attributed to Ba^2+^ was found in the selectivity filter at the K^+^ site S3, and two additional densities attributable to K^+^ ions at S2 and S4. This Ba^2+^ explains blockade, which was functionally observed more than four decades ago (Vergara and Latorre, 1983). Further, through a series of insightful experiments assessing the influence of intracellular and extracellular K^+^ on the kinetics of Ba^2+^ blockade, it was proposed the presence of K^+^ lock-in sites (Neyton and Miller, 1988a; b). In the High-Barium structure, K^+^ at S2 would be consistent with the external lock-in site while K^+^ ions at S4 and the intracellular entrance of the selectivity filter would be consistent with the internal lock-in site. On the other hand, eight Ba^2+^ ions were found within the gating ring, occupying the four high-affinity binding sites of the RCK1 domains and the four sites of the Ca^2+^ bowls (RCK2). These Ba^2+^ ions explain activation of BK channels. Similarly, in the Low-Barium structure, there were eight densities in the gating ring that are attributed to Ba^2+^ ions, but none at the selectivity filter. These results are consistent with experimental observations where Ba^2+^-activation at low Ba^2+^ concentrations precede Ba^2+^ block (Zhou et al., 2012; Miranda et al., 2016), presumably because the apparent affinity of Ba^2+^ at the selectivity filter is lower than at the high affinity binding sites of the gating ring. Why is it that we observed eight densities within the gating ring while functional work has shown that Ba^2+^ activates BK channels selectively through the Ca^2+^ bowl (Zhou et al., 2012; Miranda et al., 2016)? Even though a definitive answer is beyond the information provided by our structures, they both represent different stages of channel activation. Yet, the differences in the Ba^2+^ bound high affinity divalent sites between our structures were exclusively observed in the Ca^2+^ bowl site, suggesting that this site is readily sensitive to the changes in Ba^2+^ concentrations exposed by our experimental conditions.

Even though the selectivity filters from homologous K^+^ channels are very similar, divalent ions can occupy different sites (Jiang and MacKinnon, 2000; Guo et al., 2014; Lam et al., 2014; Rohaim et al., 2020). Although poorly understood, these differences can sometimes be associated with experimental conditions like the presence or absence of K^+^ combined with co-crystallization or soaking with Ba^2+^. For example, in crystal structures of the membrane-spanning domain of MthK K^+^ channels, a bacterial relative of BK channels, Ba^2+^ is found predominantly in the S3 and S4 in the presence of K^+^ ions, while in the presence of Na^+^, Ba^2+^ is found at S2 (Guo et al., 2014). Recently, it was demonstrated that Ba^2+^ is an open channel blocker of KcsA channels, and a divalent ion at the selectivity filter occupying the K^+^ site S4 has been reported (Rohaim et al., 2020). In *Aplysia* Slo1 channels, however, Ba^2+^ was found at site S3 in the High-Barium structure and K^+^ ions at sites S2 and S4, making High-Barium the first structure with this ion distribution.

There are three conformational changes between our structures that merit some discussion in the context of previous work. First, the expansion at the N-terminus of the gating ring between Cα atoms of Lys320 from opposite subunits is smaller than those observed previously (Hite et al., 2017; Tao et al., 2017). Nonetheless, in both cases the direction of the change is the same from a less to a more activated state of the channel. In addition, the absolute value of this distance in the 10 mM Ca^2+^/10 mM Mg^2+^ structure is 52.2 Å, larger than in the High-Ba^2+^ structure (44.7 Å), even though both models presumably represent their own maximal channel activation. This could be explained by the bend at Gly302 in the Ca^2+^ and Mg^2+^structural model, which is not present in ours. It is tempting to speculate that this bend at Gly302 is also responsible for the functional differences in the activation strength between Ca^2+^ and Ba^2+^ observed in human Slo1 (Zhou et al., 2012). Second, in our High-Ba^2+^ structure we observed a displacement of the βC strand containing Glu388, towards the position found in the Ca^2+^/Mg^2+^ structure (Hite et al., 2017; Tao et al., 2017). These results indicate that the final position of Glu388 when coordinating Mg^2+^ is not entirely induced by the presence of Mg^2+^. In addition, they are consistent with previous functional work showing that open mouse BK channels have higher apparent Mg^2+^ affinity than those closed (Shi and Cui, 2001). Third, the Asp907 side chain faces into the binding pocket in the High-Ba^2+^ structure while facing outward in the Low-Ba^2+^ structure. This same trend was also observed between the 10 mM Ca^2+^/10 mM Mg^2+^ and the 1 mM EDTA structures (Hite et al., 2017; Tao et al., 2017), indicating that the “face-in ↔ face-out” dynamics of Asp907 are not strictly dependent on the presence or complete absence of Ba^2+^. These dynamics do reveal a flexible corridor connecting the two high affinity binding sites as described here (Supplementary Video S1). These rearrangements have also been observed previously with *Aplysia* Slo1 (Hite et al., 2017; Tao et al., 2017) and in human Slo1 (Tao and MacKinnon, 2019), even though the latter has important differences in the amino acid sequences of the RCK1/RCK2 corridor, suggesting common dynamics among BK channels and different divalent ion species involved.

## Data Availability

The datasets presented in this study can be found in online repositories. The repositories and accession numbers can be found below: https://www.wwpdb.org/, 7RJT and 7RK6; https://www.ebi.ac.uk/emdb/, EMD-24490 and EMD-24493.
