# Supplementary material for: Structural bases for blockade and activation of BK channels by Ba2+ ions
Source: Front Mol Biosci. 2024 Sep 17;11:1454273. doi: 10.3389/fmolb.2024.1454273 (PMC11445749; doi:10.3389/fmolb.2024.1454273)
Supplement: Supplementary file 1 [file DataSheet2.PDF]

Supplementary Materials.

Structural bases for blockade and activation of BK channels by Ba^{2+} ions.

Shubhra Srivastava, Pablo Miranda, Teresa Giraldez, Jianghai Zhu, Raul E. Cachau and Miguel Holmgren.

	Low Barium	High Barium
**Reconstruction**		
**Particles**	973,760	594,822
**Resolution (Å)**	2.91	2.94
**Cell Dimensions**		
a,b,c (Å)	340.5,340.5,340.5	292.9,292.9,292.9
α,β,χ (°)	90,90,90	90,90,90
**Refinement**		
**No. of Residues**	3564	3608
**RMS Bond Length (Å)**	0.003	0.002
**RMS Bond Angle (°)**	0.523	0.478
**Space Group**	P1	P1
**Ramachandran Plot**		
Favored	95.7	97.4
Allowed	4.3	2.6
Outliers	0.0	0.0
**Molprobity**		
Clash Score	9.6	5.0
Rotamer Outlier (%)	0.0	0.0
Overall Score	1.8	1.4
$\mathbf{CC}_{mask}$	0.79	0.79
**PDB**	7RK6	7RJT
**EMD**	24493	24490

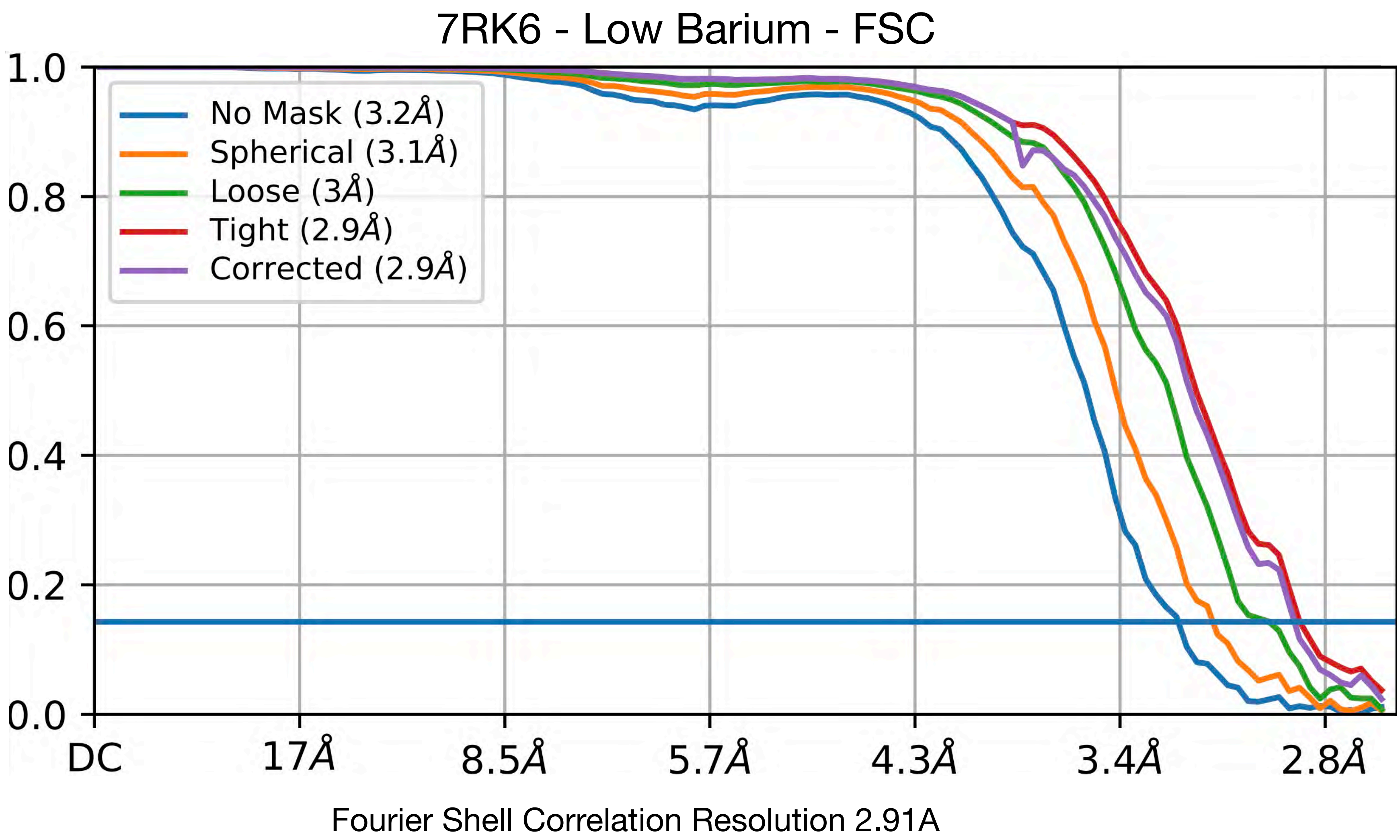
7RK6 - Low Barium - FSC
No Mask (3.2Å)
Spherical (3.1Å)
Loose (3Å)
Tight (2.9Å)
Corrected (2.9Å)
1.0
0.8
0.6
0.4
0.2
0.0
DC
17Å
8.5Å
5.7Å
4.3Å
3.4Å
2.8Å
Fourier Shell Correlation Resolution 2.91A

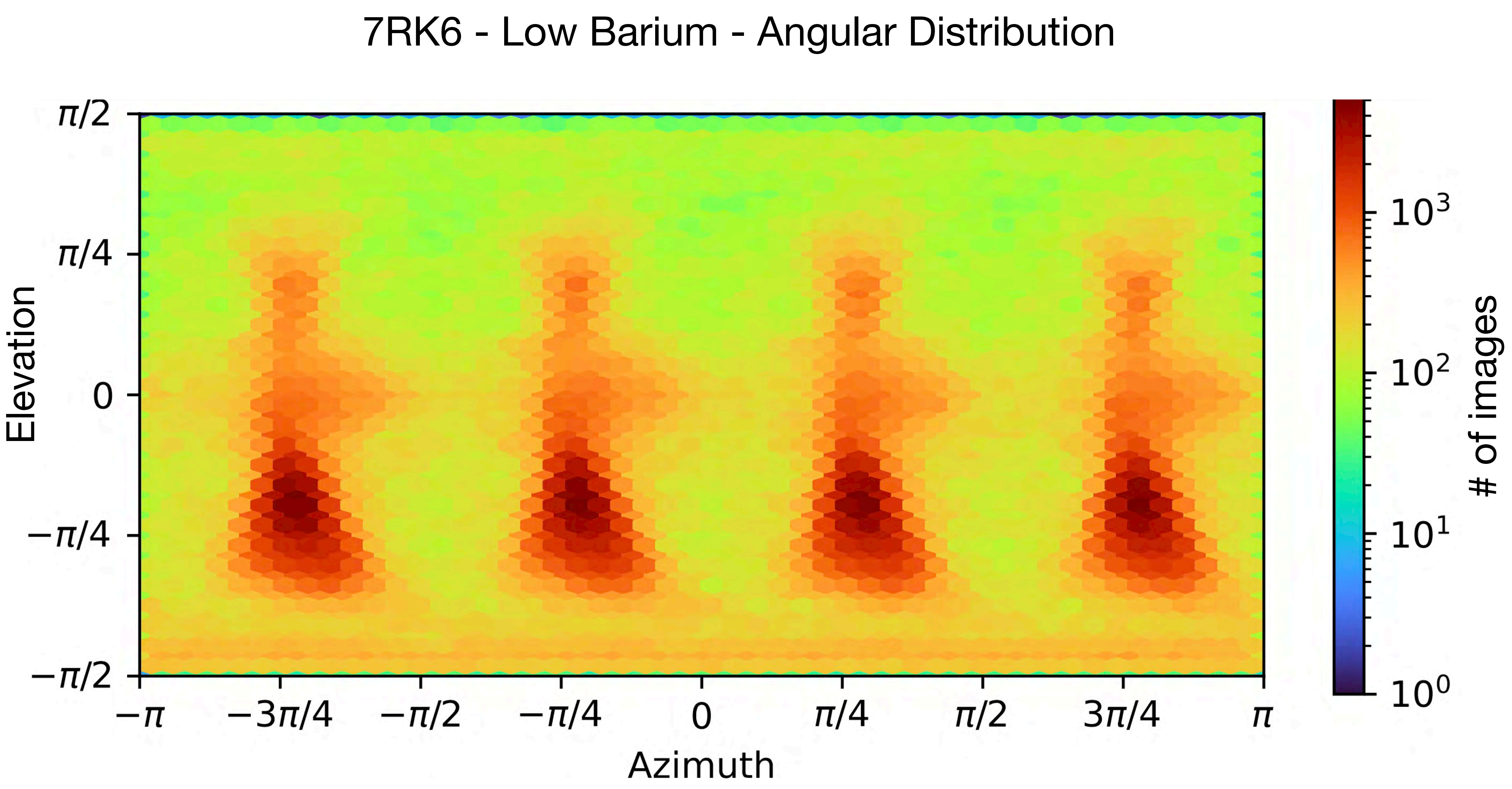
7RK6 - Low Barium - Angular Distribution
Elevation
π/2
π/4
0
−π/4
−π/2
−π
−3π/4
−π/2
−π/4
0
π/4
π/2
3π/4
π
Azimuth
10^0
10^1
10^2
10^3
# of images

7RK6 - Low Barium - Local Resolution

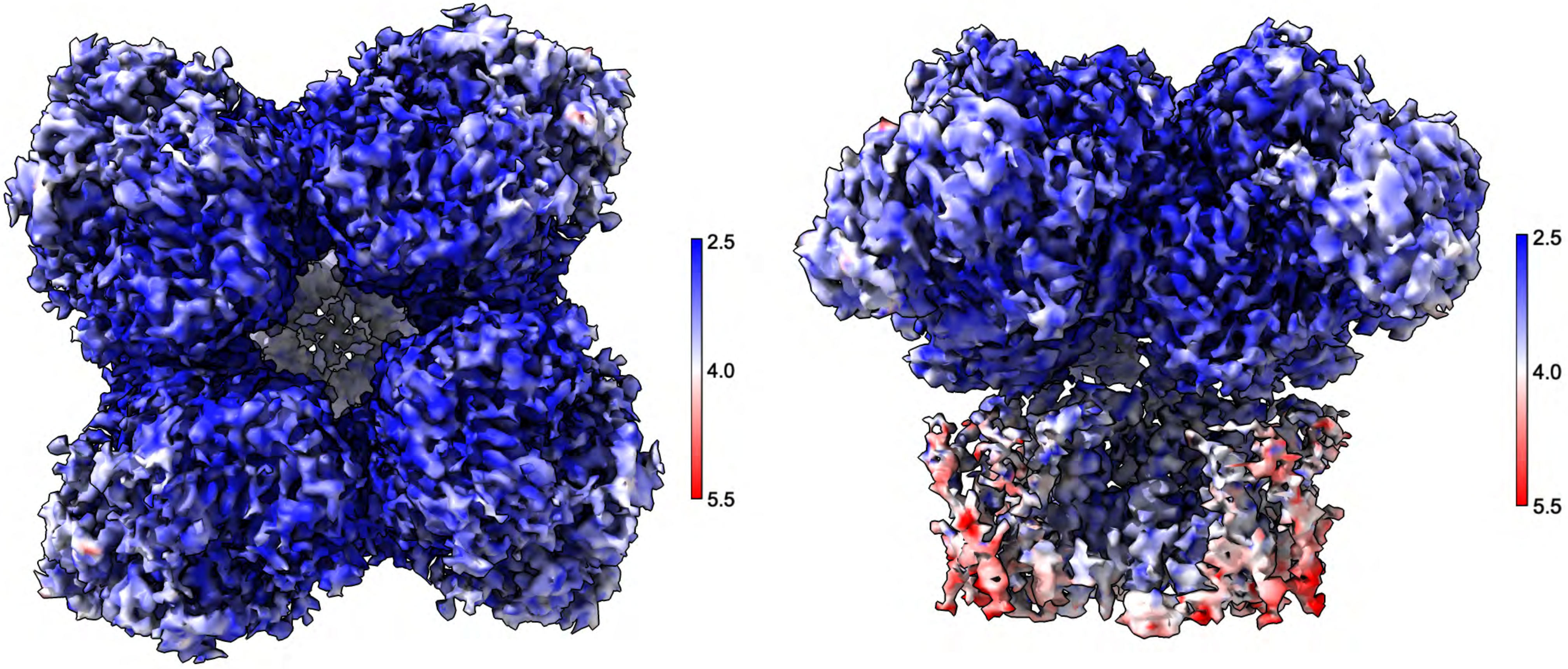

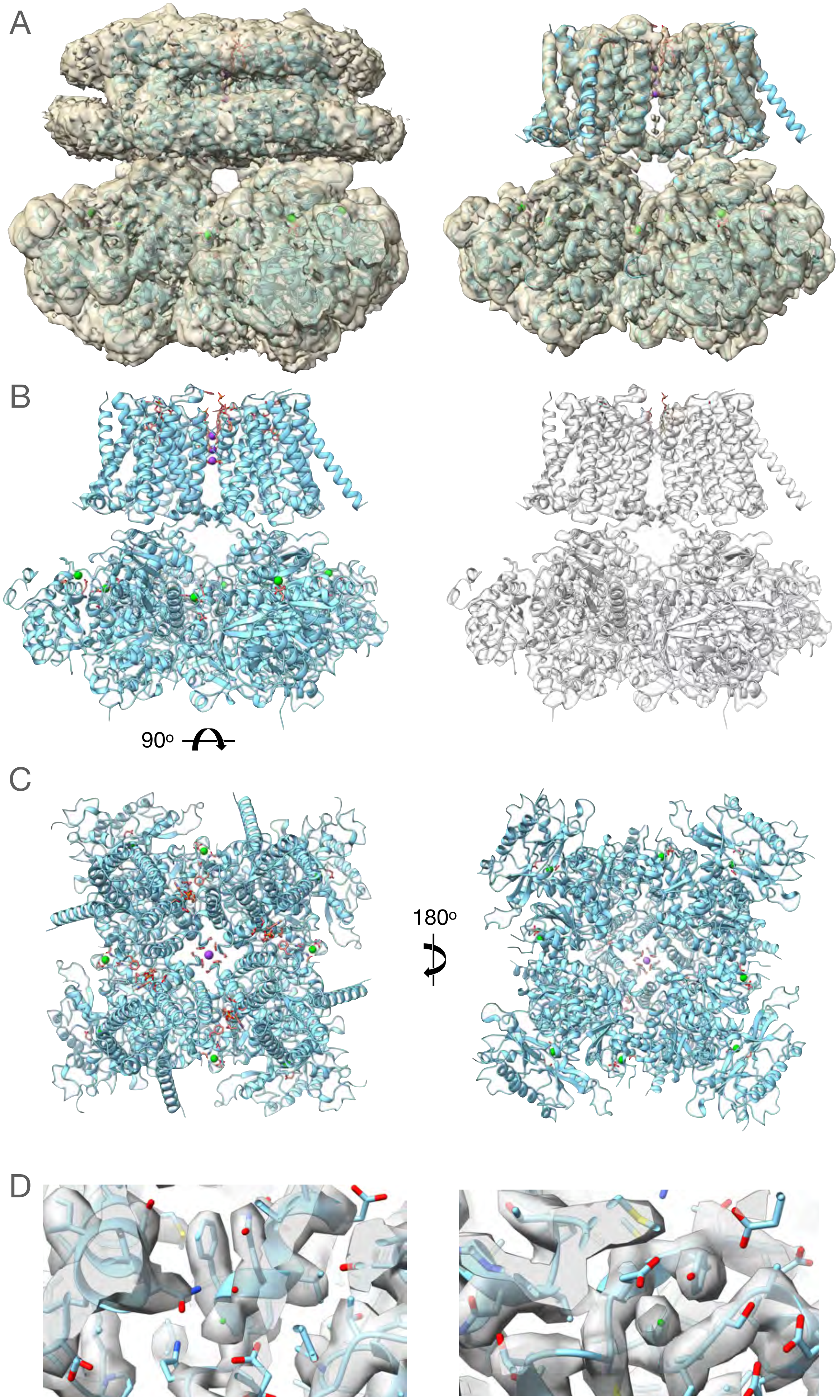
A
B
90o
C
180o
D

Low Barium Structure. Previous Figure. A) Structural model embedded in the electronic density map. On the right, the electronic densities attributed to the nanodisc were removed. B) Left, structural model showing the location of K^{+} (purple) and Ba^{2+} (green) ions, as well as the side chains of amino acids involved in the binding of ions and lipids. Right, structural model showing the location of lipids. C) Extracellular (left) and intracellular (right) views, respectively. D) Electronic densities and local structural models of the two high affinity Ca^{2+} binding sites with divalent ions bound: Ca^{2+} bowl site (left), RCK1 site (right).

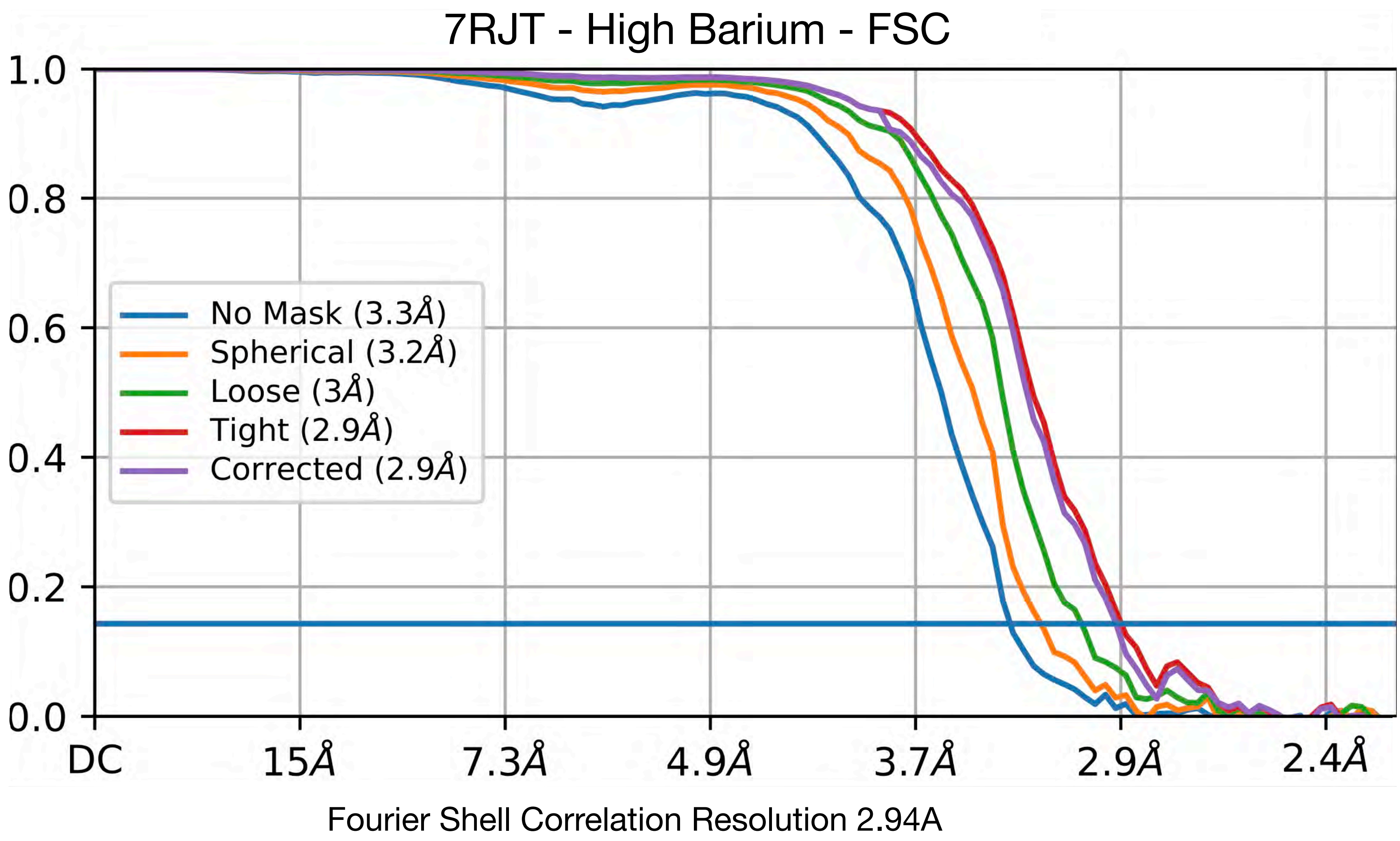
7RJT - High Barium - FSC
1.0
0.8
0.6
0.4
0.2
0.0
No Mask (3.3Å)
Spherical (3.2Å)
Loose (3Å)
Tight (2.9Å)
Corrected (2.9Å)
DC
15Å
7.3Å
4.9Å
3.7Å
2.9Å
2.4Å
Fourier Shell Correlation Resolution 2.94A

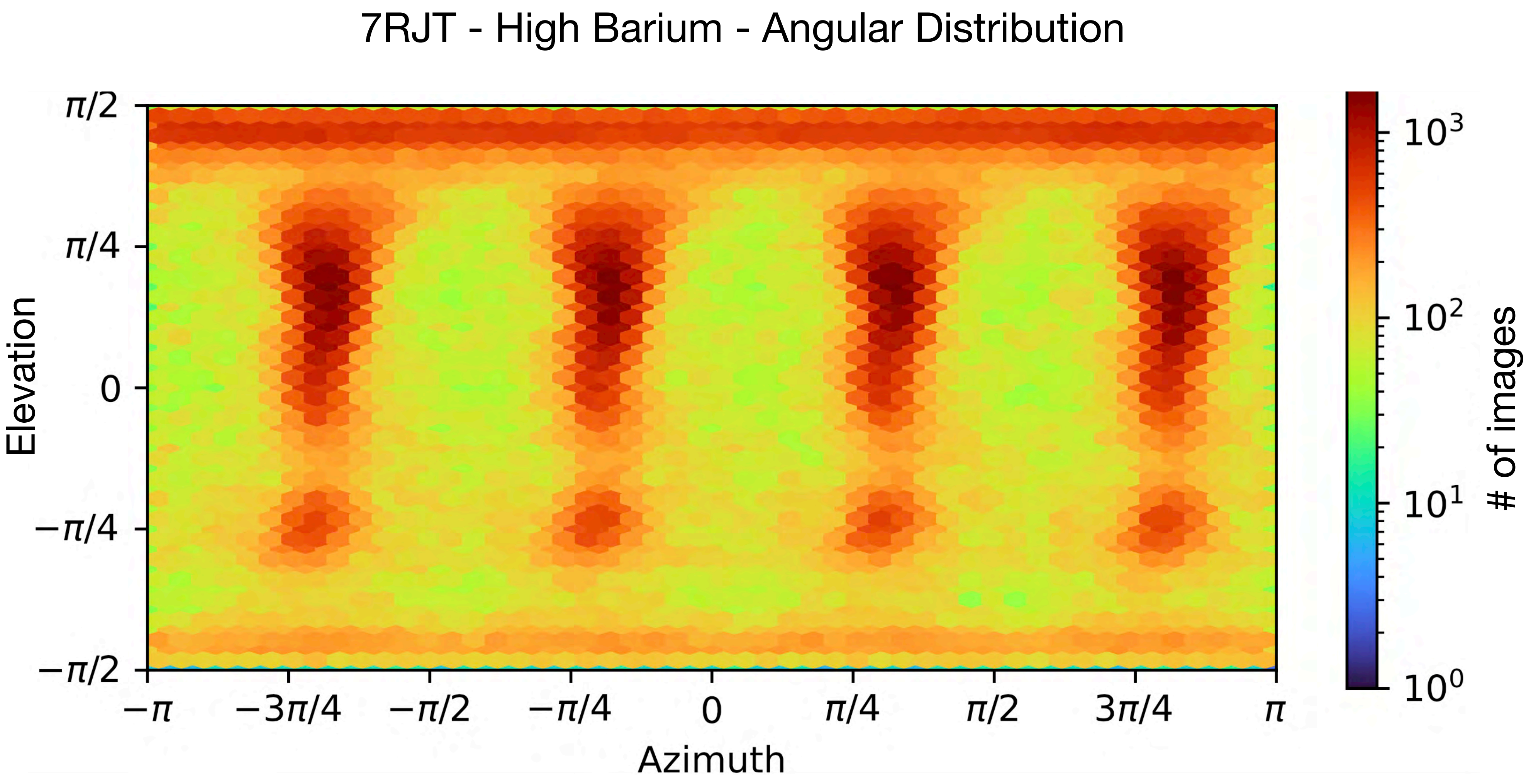
7RJT - High Barium - Angular Distribution
Elevation
π/2
π/4
0
−π/4
−π/2
−π
−3π/4
−π/2
−π/4
0
π/4
π/2
3π/4
π
Azimuth
10^3
10^2
10^1
10^0
# of images

7RJT - High Barium - Local Resolution

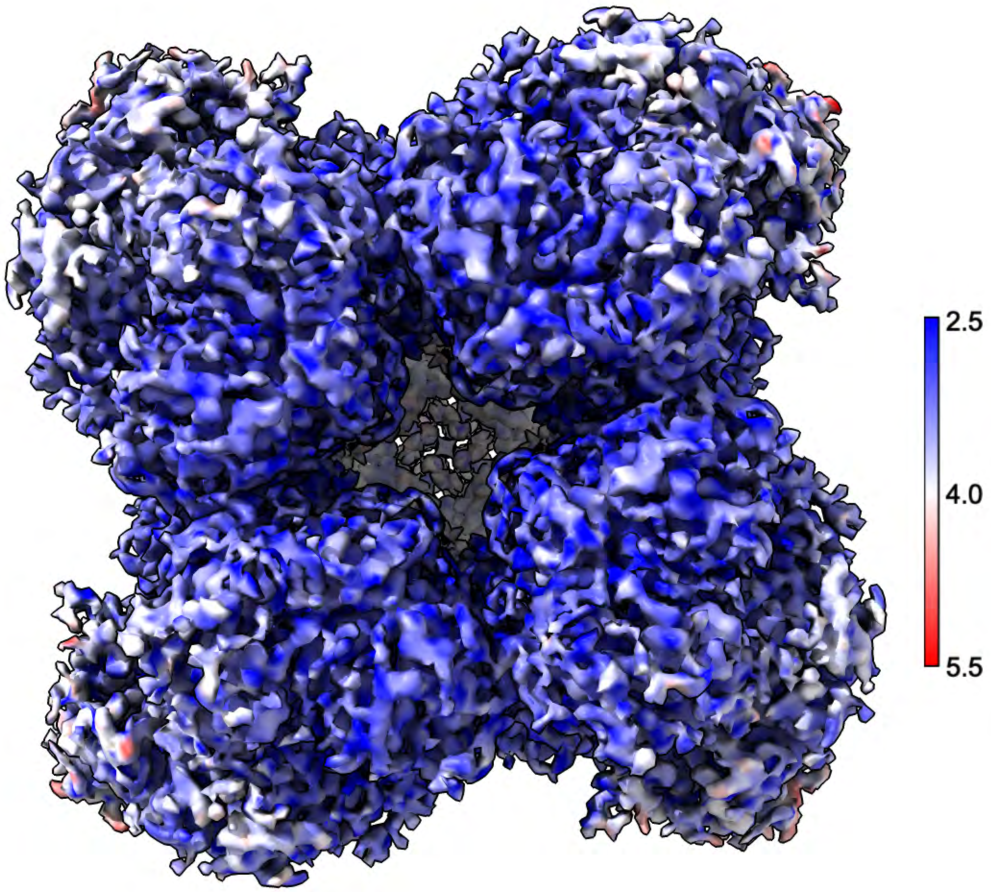

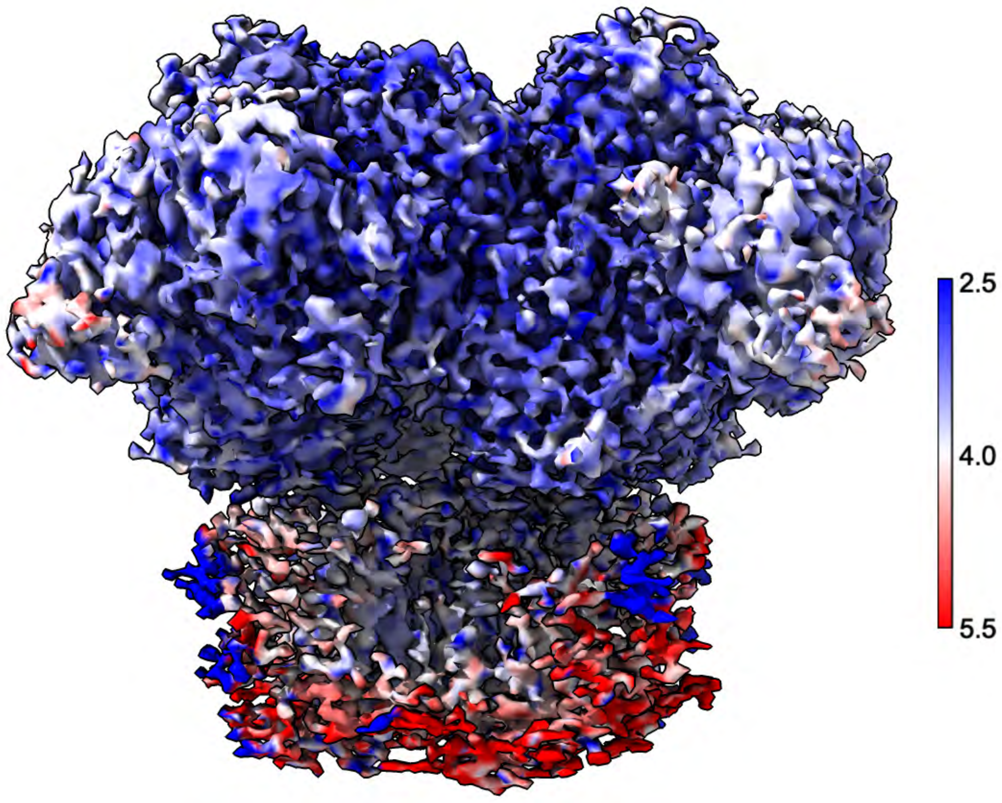

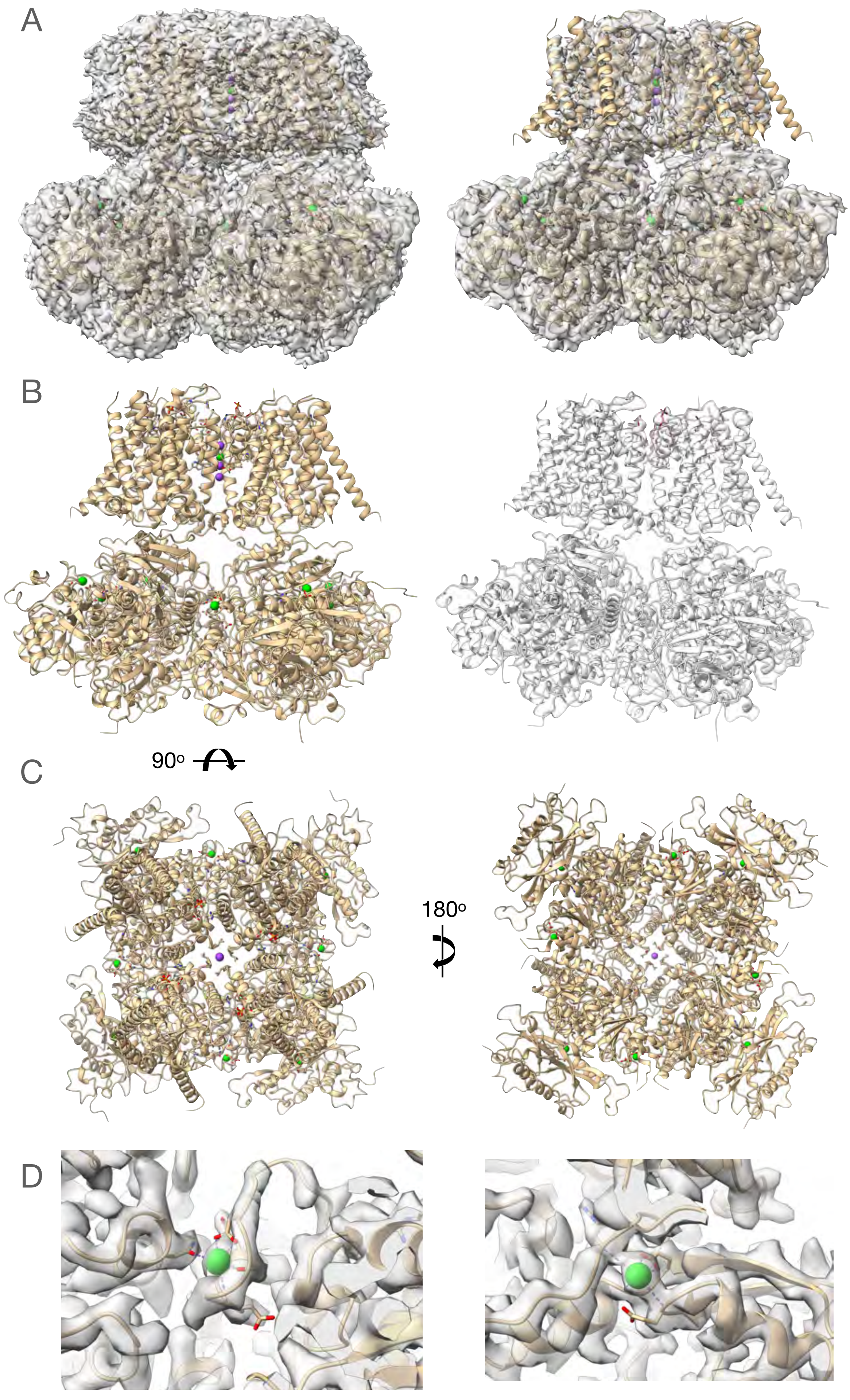
A
B
90°
C
180°
D

High Barium Structure. Previous Figure. A) Structural model embedded in the electronic density map. On the right, the electronic densities attributed to the nanodisc were removed. B) Left, structural model showing the location of K^+ (purple) and Ba^{2+} (green) ions, as well as the side chains of amino acids involved in the binding of ions and lipids. Right, structural model showing the location of lipids. C) Extracellular (left) and intracellular (right) views, respectively. D) Electronic densities and local structural models of the two high affinity Ca^{2+} binding sites with Ba^{2+} ions bound: Ca^{2+} bowl site (left), RCK1 site (right).

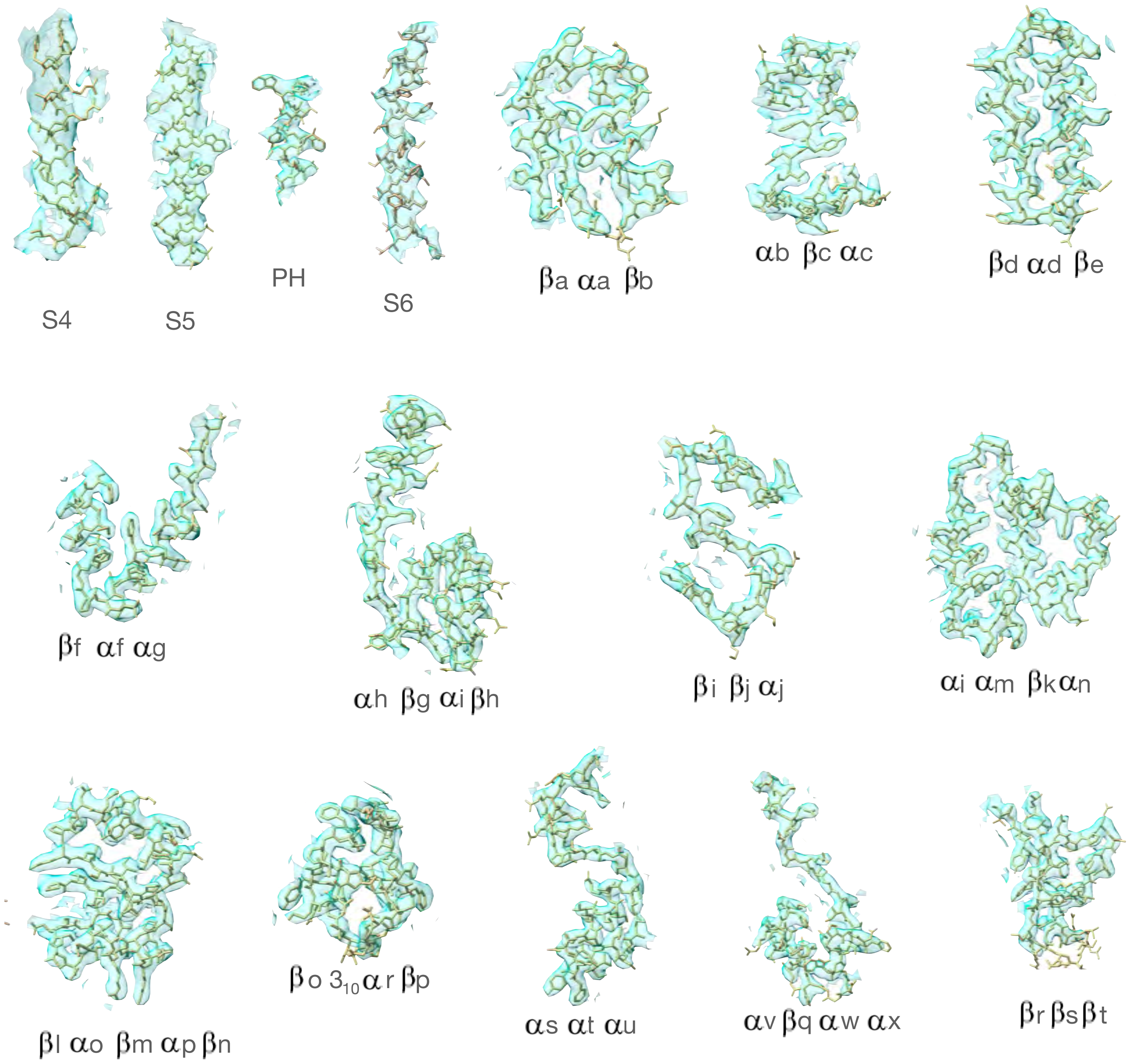
S4
S5
PH
S6
βa αa βb
αb βc αc
βd αd βe
βf αf αg
αh βg αi βh
βi βj αj
αi αm βk αn
βl αo βm αp βn
βo 3_{10} αr βp
αs αt αu
αv βq αw αx
βr βs βt

Previous Figure, examples of local density maps and corresponding structural models throughout the transmembrane and gating ring regions.

A

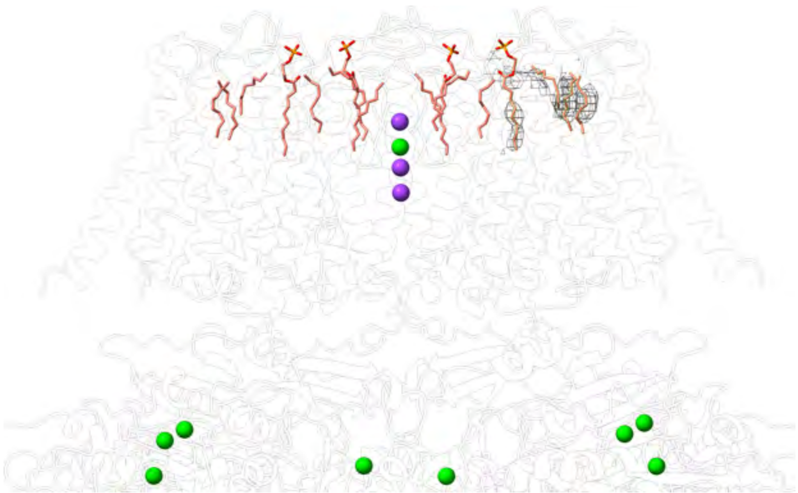

B

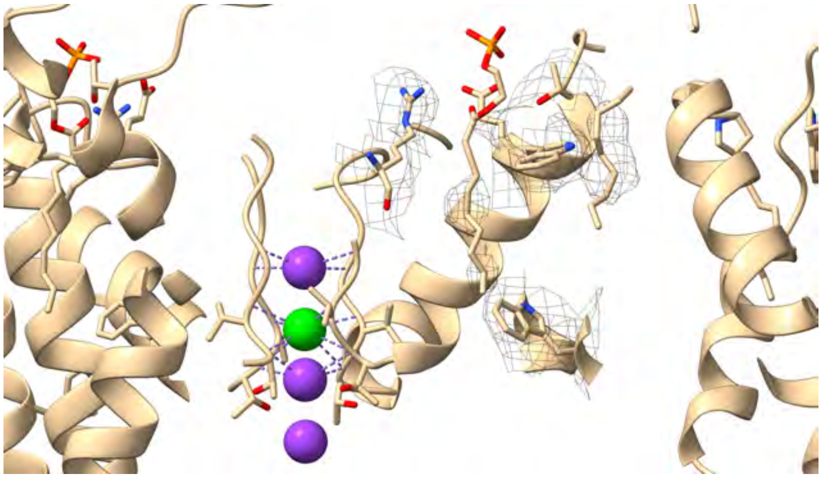

C

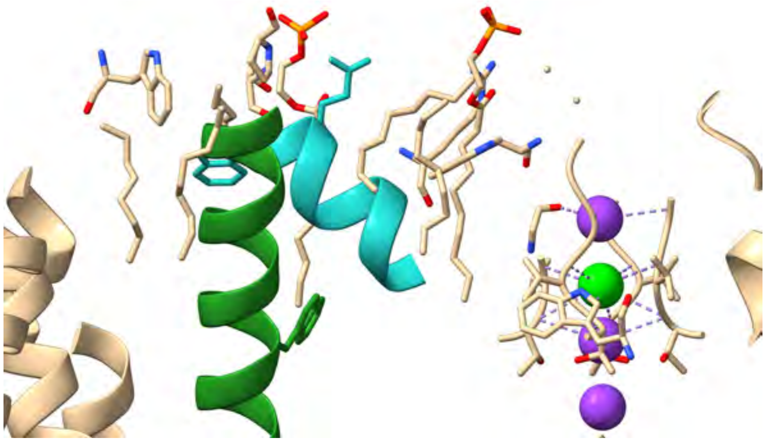

Electron densities attributable to lipids are observed in the High Barium structure. A) A general view of the structure with the densities attributable to lipids shown. B) Detailed view of the region where electron densities are observed. Putative interactions between the lipid head and charged side chains suggest an ionic interaction. The chemical identity of the lipids cannot be determined due to the low resolution of the map in this region. C) Lipids hydrophobic tails cluster in a Tryptophan-rich region flanked by S5 and PH helices from neighboring chains, suggesting a possible functional role for lipid-protein interactions near the selectivity filter.
