# Supplementary material for: Structural bases for blockade and activation of BK channels by Ba2+ ions
Source: Front Mol Biosci. 2024 Sep 17;11:1454273. doi: 10.3389/fmolb.2024.1454273 (PMC11445749; doi:10.3389/fmolb.2024.1454273)
Supplement: Supplementary file 2 [file DataSheet1.pdf]

Supplementary Materials.

Structural bases for blockade and activation of BK channels by Ba^{2+} ions.

Shubhra Srivastava, Pablo Miranda, Teresa Giraldez, Jianghai Zhu, Raul E. Cachau and Miguel Holmgren.

Expression and purification

The gene fragment encoding the full-length Slo1 from *Aplysia californica* was expressed as a fusion construct with a C-terminal GFP/rho-1D4 tag cleavable by precision protease in Sf9 cells (Hite et al., 2017;Tao et al., 2017). The baculovirus was generated in Sf9 cells following the standard Bac-to-Bac system protocol. In brief, sub-confluent Sf9 cells were transfected with recombinant Slo1 bacmid DNA using Cellfectin II transfection reagent. After a four-day incubation period, the supernatant from the cell culture was collected, yielding the P1 recombinant baculovirus. Subsequent rounds of infection with P1 virus in Sf9 cells allowed for the generation of P2 and P3 virus stocks, amplifying the viral load for subsequent protein expression. Sf9 cells were cultured in SFM III Expression Medium for large-scale protein expression. At a cell density of $2x10^6$ cells per ml, infection was performed with recombinant baculovirus and incubated at 27 °C with agitation. Following a 48-hour incubation period post-infection, the cells were harvested by centrifugation. All subsequent purification steps were meticulously carried out at a low temperature of 4°C to maintain protein stability and integrity.

The cell pellet was re-suspended and lysed by osmotic shock in a hypotonic buffer consisting of 10mM Tris-HCl pH 8.0, 3mM dithiothreitol (DTT), 1mM EDTA, and protease inhibitors pepstatinA (0.1μg/ml), aprotinin (1μg/ml), leupeptin (1μg/ml), soy trypsin inhibitor (0.1μg/ml), phenylmethylsulphonyl fluoride (0.2mM), benzamidine (1mM) and 4-(2-aminoethyl) benzenesulfonyl fluoride hydrochloride (0.1mg/ml). After lysis, cell membranes were collected by centrifugation at 30000g for 30 min. The pellet was homogenized in a buffer containing 20 mM Tris-HCl pH 8.0, 320mM KCl, 15mM $BaCl_2$, 5mM EGTA, and all protease inhibitors added previously. n-Dodecyl-β-D Maltopyranoside (DDM) and Cholesterol Hemi Succinate (CHS) were added to the cell membrane suspension to achieve a 1% and 0.2% (w/v), respectively. Then, the suspension was stirred gently for 1 hr, followed by ultracentrifugation at 45000g for 30 min. Clarified supernatant was added to a GFP nanobody-conjugated affinity resin pre-equilibrated with buffer composed of 20mM Tris-HCl pH 8.0, 320mM KCl, 15mM $BaCl_2$, 5mM EGTA, pepstatinA (0.1μg/ml), aprotinin (1μg/ml) and soy trypsin inhibitor (0.1μg/ml). The suspension was mixed by inversion for 2 hrs. The resin was collected by a 5 min. slow speed (100g) centrifugation, transferred into a gravity column, and washed 2 times with 10X column volume with washing buffer (WB) containing 20mM Tris-HCl pH 8.0, 320mM KCl, 15mM $BaCl_2$, 5mM EGTA, pepstatinA (0.1μg/ml), aprotinin (1μg/ml), soy trypsin inhibitor (0.1μg/ml) and 0.2%DDM/0.04%CHS. On the column, cleavage of the protein was achieved by incubating the resin with WB buffer supplemented with 3C protease to an approximate molar ratio of 20:1, followed by overnight incubation at 4°C under slow rocking motion. Cleaved protein was collected, concentrated, and ran through a Superose 6 column equilibrated in SEC buffer (20 mM Tris-HCl pH 8.0, 320mM KCl, 15mM $BaCl_2$, 5mM EGTA, 20mM DTT, 2mM TCEP, 0.025% DDM, pepstatin A (0.1μg/ml) and aprotinin (1μg/ml). The fractions containing tetrameric *Aplysia* Slo1 channels were concentrated to approximately 7-10 mg/ml using an Amicon Ultra centrifugal

filter (100-kDa MW cutoff). Within the same day of purification, samples were further used for incorporation into lipid nanodiscs.

Reconstitution of Channel in nanodiscs

Membrane scaffold protein MSP1E3D1 was expressed and purified from *Escherichia coli* as previously described (Inagaki et al., 2013), and *Aplysia* Slo1 tetramers were incorporated into lipid nanodiscs following the published protocol (Ritchie et al., 2009). Lipid stock was obtained from Avanti Polar Lipids. Initially, Lipids were evaporated to remove their chloroform solvent. Dry lipid films were resuspended in water and detergent (DDM) to a final DDM concentration of 25mM. The lipid/detergent mixture was sonicated until the solution became nearly transparent and aliquots were frozen at −80°C.

Slo1, MSP1E3D1, and lipid were mixed at a molar ratio of 1:2:85, respectively, and incubated on ice for 30 min. Detergent was removed by adding Bio-Beads SM2 to 20mg/ml, followed by gentle agitation for 3 hours. The Bio-Beads were then removed by passage through a PolyPrep column, and the flow-through was centrifuged (10,000g) before size-exclusion chromatography. Ultracentrifuged samples were purified by size-exclusion chromatography on a Superose 6 Increase 10/300 GL column, equilibrated with nanodisc SEC buffer (20 mM Tris-HCl pH 8.0, 320mM KCl, 15mM $BaCl_2$, 5mM EGTA, 20mM DTT, 2mM TCEP, pepstatin A (0.1μg/ml) and aprotinin (1μg/ml). Fractions containing the nanodisc/channel complexes were collected and concentrated using a centrifugal filter unit (100 kDa molecular weight cutoff). The samples were transferred on ice to the Cryo-EM Facility, Center for Molecular Microscopy (CMM), Center for Cancer Research (CCR), National Cancer Institute (NCI), Leidos Biomedical Research, Inc., where Cryo-EM grids were prepared within 3 hrs from the last step of purification.

Low-Barium structure

Here, the sample was treated similar as previously described in the last two sections, except the sample was exposed to 40 mM free $BaCl_2$ in all steps but the last, in which the Superose 6 column was equilibrated in nanodisc SEC buffer without $BaCl_2$. Using a divalent sensing electrode (Calcium electrode 9720BNWP ThermoFisher), we estimated that the fractions containing nanodisc/channel complexes has ~1.4 mM free Ba^{2+}. Assuming a similar dilution factor, the EGTA concentration should be ~0.17 mM. To confirm electrophysiologically, we diluted the solution from the SEC fractions containing nanodisc/channel complexes 100X with a solution containing 20mM Tris-HCl pH 8.0, 320mM KCl and 0.17mM EGTA. Further, to facilitate gigaseal formation, the solution in the pipette contained 20mM Tris-HCl pH 8.0, 320mM KCl, 1mM $CaCl_2$ and 1mM $MgCl_2$. With these conditions, the 100X dilution was able to activate Aplysia BK channels followed by blockade, like the results shown in Fig. 1 with 10 μM Ba^{2+}. This study shed new light on the paradoxical role of Ba^{2+}. The two structures here reported show that the binding of the Ba^{2+} to the selectivity filter is more concentration dependent compared to the RCK1/RCK2 binding sites. A quantitative assessment of the ion occupancy in the CryoEM Low-Ba^{2+} structure is challenging due to the limitations of current CryoEM technologies to elucidating the identity of single-density peaks. Therefore, attributing the identity of the density peak at the selectivity filter in the Low-Ba^{2+}

structure is not warranted.

Hite, R.K., Tao, X., and Mackinnon, R. (2017). Structural basis for gating the high-conductance Ca^{2+}-activated K^+ channel. *Nature* 541**,** 52-57.

Inagaki, S., Ghirlando, R., and Grisshammer, R. (2013). Biophysical characterization of membrane proteins in nanodiscs. *Methods* 59**,** 287-300.

Ritchie, T.K., Grinkova, Y.V., Bayburt, T.H., Denisov, I.G., Zolnerciks, J.K., Atkins, W.M., and Sligar, S.G. (2009). Chapter 11 - Reconstitution of membrane proteins in phospholipid bilayer nanodiscs. *Methods Enzymol* 464**,** 211-231.

Tao, X., Hite, R.K., and Mackinnon, R. (2017). Cryo-EM structure of the open high-conductance Ca^{2+}-activated K^+ channel. *Nature* 541**,** 46-51.

CryoEM - Methods

Samples were diluted to optimal concentrations ranging from 2 mg/ml to 8 mg/ml in SEC or ND buffer. Three microliters of each sample were applied to a Quantifoil R 1.2/1.3 Cu 200 mesh grid that had been glow discharged for 5 seconds using a PELCO easiGlow system (Ted Pella, Inc.) at 15 mA and 0.3 mBar. Grids were then plunged into liquid ethane with a Vitrobot Mk IV (Thermo Fisher Scientific) operated at 4°C and 100% humidity, with a blot time of 2 seconds and a wait time of 5 seconds. Grids were stored in liquid nitrogen until imaging. Cryo-EM data were collected on a Titan Krios electron microscope (Thermo Fisher Scientific) operated at 300 kV and equipped with a K2 Summit direct electron detector (Gatan). Micrographs were acquired in counted mode using SerialEM (Mastronarde, 2005) v3.8 with a 3'3 multi-shot setup and a nominal magnification of 29,000', resulting in a pixel size of 0.858 Å. A total dose of 50 $e^-/Å^2$ was fractionated over 40 frames. Data quality was monitored during collection using cryoSPARC (Punjani et al., 2017) Live v3.2 preprocessing.

Motion correction, dose-fractionated weighting, and binning of collected movie data were carried out by MotionCor2 (Zheng et al., 2017) v1.4. The parameters of CTF were estimated by CTFFIND4 (Rohou and Grigorieff, 2015) v4.1. EMD-8410 was used as the template for template-matching particle-picking in RELION v3.1 (Scheres, 2012). This set of particles was classified in 2D iteratively to produce a set of 2D templates for another round of automated particle-picking. To clean up the data, extracted particles were classified in 2D for as many rounds as necessary in cryoSPARC v3.2. *Ab initio* volumes were determined in cryoSPARC with 5 classes, followed by heterogeneous refinement. High-quality particles were pooled. High-resolution 3D volume refinement was carried out in RELION, followed by postprocessing, CTF and aberration refinement, and Bayesian polishing. These "shiny" particles were transferred to cryoSPARC again for *ab initio* volume determination, homogeneous refinement, and non-uniform homogeneous refinement (Punjani et al., 2020). C4 symmetry was imposed during later refinements. Local resolution was determined by cryoSPARC.

Maps output by cryoSPARC were sharpened by PHENIX.auto_sharpen (PHENIX (Adams et al., 2010) v1.19). *Aplysia* Slo1 structure (PDBID:5tj6) was docked into maps using ChimeraX (Pettersen et al., 2021) v1.2, then refined by molecular dynamics using the ISOLDE

(Croll, 2018) v1.2 plugin. Cycles of manual model building in COOT (Emsley and Cowtan, 2004) v0.9, followed by real-space refinement in PHENIX.real_space_refine, were repeated several rounds until no noticeable improvement could be observed. The whole tetramer of Slo1, generated by a 4-fold symmetry, is used for real-space refinement, but three of the four subunits are the exact symmetric copies of the sole independent subunit. Metal ions inside the selective filter are located precisely on the 4-fold symmetry axis. MolProbity (Davis et al., 2007) v4.5 was used for model validation. Figures were prepared by ChimeraX. FSC and angular distribution plots were directly output by cryoSPARC. Local resolution figures were generated by ChimeraX.

Adams, P.D., Afonine, P.V., Bunkoczi, G., Chen, V.B., Davis, I.W., Echols, N., Headd, J.J., Hung, L.W., Kapral, G.J., Grosse-Kunstleve, R.W., Mccoy, A.J., Moriarty, N.W., Oeffner, R., Read, R.J., Richardson, D.C., Richardson, J.S., Terwilliger, T.C., and Zwart, P.H. (2010). PHENIX: a comprehensive Python-based system for macromolecular structure solution. *Acta Crystallogr D Biol Crystallogr* 66**,** 213-221.

Davis, I.W., Leaver-Fay, A., Chen, V.B., Block, J.N., Kapral, G.J., Wang, X., Murray, L.W., Arendall, W.B., 3rd, Snoeyink, J., Richardson, J.S., and Richardson, D.C. (2007). MolProbity: all-atom contacts and structure validation for proteins and nucleic acids. *Nucleic Acids Res* 35**,** W375-383.

Emsley, P., and Cowtan, K. (2004). Coot: model-building tools for molecular graphics. *Acta Crystallogr D Biol Crystallogr* 60**,** 2126-2132.

Mastronarde, D.N. (2005). Automated electron microscope tomography using robust prediction of specimen movements. *J Struct Biol* 152**,** 36-51.

Pettersen, E.F., Goddard, T.D., Huang, C.C., Meng, E.C., Couch, G.S., Croll, T.I., Morris, J.H., and Ferrin, T.E. (2021). UCSF ChimeraX: Structure visualization for researchers, educators, and developers. *Protein Sci* 30**,** 70-82.

Punjani, A., Rubinstein, J.L., Fleet, D.J., and Brubaker, M.A. (2017). cryoSPARC: algorithms for rapid unsupervised cryo-EM structure determination. *Nat Methods* 14**,** 290-296.

Punjani, A., Zhang, H., and Fleet, D.J. (2020). Non-uniform refinement: adaptive regularization improves single-particle cryo-EM reconstruction. *Nat Methods* 17**,** 1214-1221.

Rohou, A., and Grigorieff, N. (2015). CTFFIND4: Fast and accurate defocus estimation from electron micrographs. *J Struct Biol* 192**,** 216-221.

Scheres, S.H. (2012). RELION: implementation of a Bayesian approach to cryo-EM structure determination. *J Struct Biol* 180**,** 519-530.

Zheng, S.Q., Palovcak, E., Armache, J.P., Verba, K.A., Cheng, Y., and Agard, D.A. (2017). MotionCor2: anisotropic correction of beam-induced motion for improved cryo-electron microscopy. *Nat Methods* 14**,** 331-332.
